# Emerging Challenges of Preclinical Models of Anti-tumor Immunotherapeutic Strategies Utilizing Vγ9Vδ2 T Cells

**DOI:** 10.3389/fimmu.2020.00992

**Published:** 2020-05-22

**Authors:** Noémie Joalland, Emmanuel Scotet

**Affiliations:** ^1^Université de Nantes, INSERM, CNRS, CRCINA, Nantes, France; ^2^LabEx IGO “Immunotherapy, Graft, Oncology”, Nantes, France

**Keywords:** human Vγ9Vδ2 T lymphocytes, cancer, functions, immunotherapy, preclinical models

## Abstract

Despite recent advances, the eradication of cancers still represents a challenge which justifies the exploration of additional therapeutic strategies such as immunotherapies, including adoptive cell transfers. Human peripheral Vγ9Vδ2 T cells, which constitute a major transitional immunity lymphocyte subset, represent attractive candidates because of their broad and efficient anti-tumor functions, as well as their lack of alloreactivity and easy handling. Vγ9Vδ2 T cells act like immune cell stress sensors that can, in a tightly controlled manner but through yet incompletely understood mechanisms, detect subtle changes of levels of phosphorylated metabolites of isoprenoid synthesis pathways. Consequently, various anti-tumor immunotherapeutic strategies have been proposed to enhance their reactivity and cytotoxicity, as well as to reduce the deleterious events. In this review, we expose these advances based on different strategies and their validation in preclinical models. Importantly, we next discuss advantages and limits of each approach, by highlighting the importance of the use of relevant preclinical model for evaluation of safety and efficacy. Finally, we propose novel perspectives and strategies that should be explored using these models for therapeutic improvements.

## Biology and Anti-Tumor Functions OF Human Vγ9Vδ2 T Cells

The complex immune system orchestrates with molecular and cellular components that act concomitantly or sequentially to sense and to eliminate potential exogenous and endogenous threats. In vertebrates, the innate immunity is the first germline-encoded and older evolutionary defense strategy that contributes to a rapid, but poorly specific, reactivity that also remains essential for recruiting immune cells and establishing a physical and chemical barrier. A major hallmark of the adaptive immunity, that is associated to immunological memory and next takes place after this initial process, is the extreme specificity against particular antigenic structures. This slower, but highly potent, immune reactivity axis is made up of the two mainT and B lymphocyte effector subsets. Recently, a growing class of additional cellular contributors, that express either TCR (*T Cell Receptor*) or BCR (*B Cell Receptor*) molecules and share phenotypical and functional characteristics from both systems has been identified. They have been differentially named as transitional immunity, unconventional or innate-*like* effectors. Most well-described cell subsets that fall at this interface between innate and adaptive immunities are NKT (*Natural Killer T*), MAIT (*Mucosal Associated Invariant T*) and γδ T cells (1). This latter T cell subset expresses a heterodimeric TCR composed of γ and δ chains, named as opposed to α and β chains, associated to the CD3 signaling complex (2, 3). It is important to note that, although γδ T cell subsets are present in most vertebrates, there is little conservation of γ and δ TCR chains and reactivities between species. This has been evidenced by genetic studies indicating a high heterogeneity between murine and primate TCR genes (4, 5).

For the sake of clarity, we propose to focus this present review on human γδ T cell biology, and more precisely, the human Vγ9Vδ2 T cell subset, as well as their therapeutic targeting in preclinical cancer models. The γδ TCR is composed from a limited number of γ and δ chains (<15 Vγ and Vδ gene segments) but this low combinatorial diversity is efficiently counterbalanced by both an elevated junctional diversity and pairing assembly restrictions to finally produce an extremely diverse γδ TCR. In humans, γδ T cells are distributed into four major subsets, which have been identified according to the expression of δ chain segments, as Vδ1^+^, Vδ2^+^, Vδ3^+^, and Vδ5^+^ populations (6). Following ontogeny, most γδ T cell subsets naturally exhibit a preferential tropism for particular tissues/organs, through yet unclear mechanisms. As a typical example of this preferential localization, human Vγ9Vδ2 T cells [using the nomenclature of Lefranc & Rabbitts (7)] constitute a major subset in adult peripheral blood (8, 9). Vγ9Vδ2T cells represent prototypical immune sensors of cellular stress activated under various pathological contexts, such as infections and cancer. The species-specific antigenic activation of Vγ9Vδ2 T cells is a contact- and a TCR-dependent process. It is important to note that this activation is not restricted by either conventional MHC class I or II molecules, thus theoretically minimizing the risk of alloreactive reactions for allogeneic Vγ9Vδ2 T cell-therapies (e.g., GVHD, *graft vs. host disease*). This antigenic activation process implicates mandatory small phosphorylated carbohydrate metabolites (hereafter called phosphoantigens, PAg), such as IPP (*Isopentenyl PyroPhosphate*), which endogenous (MEV mammalian mevalonate pathway), or exogenous (MEP microbial pathway) expression might be altered in some pathological contexts (e.g., cancer, infections). Recent studies have shown that PAg levels are sensed through mechanisms involving both BTN2A1 and BTN3A1 butyrophilins (BTN) expressed by target cells (10–12). Accordingly, pharmacological compounds that inhibit the synthesis (i.e., statins) or the degradation (i.e., aminobisphosphonates, alkylamines) of PAg in mammalian cells can block, or induce, the antigenic activation of Vγ9Vδ2 T cells, respectively (13, 14). Importantly, this *Self*-reactive nature of Vγ9Vδ2 T cells needs to be tightly regulated by a set of various molecules [see (15) and (16), for recent reviews], such as adhesion molecules (i.e., CD54), activating (i.e., NKG2D) or inhibiting (i.e., CD94/NKG2A,) NKR (*Natural Killer Receptors*), FcR (*Fc Receptor*) (i.e., FcRγIIIA/CD16), Nectin/Nectin-*like* (i.e., CD226), TLR (*Toll-like Receptor*) (i.e., TLR4), cytokine receptors (i.e., IL (*interleukin*)-15R, IL-21R), and immune checkpoint inhibitors (i.e., PD-1, *programmed cell death protein 1*). Hence, numerous Vδ2 T cell dysfunctions in cancer indications (e.g., hypo-reactivity, exhaustion) have been associated to altered expression profiles of these molecules. In this exquisite sensing process, Vγ9Vδ2 T cells integrate activatory and inhibitory signals to rapidly deliver strong functional responses such as proliferation, cytolysis (through perforin/granzyme-, TRAIL (*TNF-related apoptosis inducing ligand*)-, CD95-pathways) and cytokines (i.e., TNF (*Tumor Necrosis Factor*)-α, IFN (*Interferon*)-γ)/chemokines (i.e., CCL3, *Chemokine (C-C motif) ligand 3*)/anti-microbial factors (i.e., granulysin)/epithelial growth factors (i.e., KGF, *Keratinocyte Growth Factor*) release. These latter functions are also linked to their capacity to help other immune effectors and induce the maturation of antigen-presenting cells, including themselves (9). Finally, as for most lymphocyte subsets, the migration of Vγ9Vδ2 T cells is tightly controlled by a set of chemoattractant factors, such as chemokine receptors. Their expression regulates Vγ9Vδ2 T cell trafficking during physiological and inflammatory conditions (e.g., CCR5, *Chemokine (C-C motif) Receptor 5*), a process that is proposed to be of particular importance for tumor addressing and infiltration *in vivo*.

For a long time, a set of compelling *in vitro* studies evidenced the natural reactivity of human Vγ9Vδ2 T cells against a broad range of human tumor cell lines and normal cells infected by a variety of viruses, parasites and bacteria (17–19). With respect to transformed cells, the range of cell lines recognized by Vγ9Vδ2 T cells, initially thought to be primarily restricted to hematopoietic tumors (20, 21), was next extended to several solid tumors, such as renal and colon carcinomas (22–24). Importantly, this vision has been next modified by the availability of aminobisphophonates (e.g., pamidronate, zoledronate) and synthetic PAg (e.g., BrHPP, *BromoHydrin Pyrophosphate*) that can further help sensitizing a broad variety of cells to Vγ9Vδ2 T cell sensing and elimination. Like for most γδ T cell subsets, *in vitro* studies showed that Vγ9Vδ2 T cells are able to directly kill target cells and express pro-inflammatory cytokines that can be also involved in the clearance of tumor cells (25, 26). Altogether, these *in vitro* observations supported a natural implication of Vγ9Vδ2 T cells in protective anti-tumor immunity. Based on initial results indicating an altered tumor growth control in TCR δ^neg^ mice (27), several *in vivo* studies showed that transferred allogeneic Vγ9Vδ2 T cells can reach and infiltrate tumor site and display a strong anti-tumor activity as evidenced by significant clinical benefits (e.g., survival, tumor growth) (28, 29). The implication of Vγ9Vδ2T cells in the anti-tumor immune reactivity is supported by the fact that infiltrating γδ T cells are considered as a favorable cancer prognosis marker for several cancers (30, 31), Vδ2 T cells infiltrating tumors were detected in various types of cancer. However, their precise physiological role might vary from one condition to another, mainly due the heterogeneity of the tumor microenvironment which can modulate their functions as well as their functional plasticity (30, 31).

## Rationale for Harnessing Vγ9Vδ2 T Cells in Cancer Immunotherapy

Human Vγ9Vδ2 T cells should be considered as attractive immune effectors of high therapeutic potential for the main following reasons:

1. Inter-individual conservation and elevated frequency in the peripheral blood of human adults;
2. Antigenic specificity linked to cell stress-associated molecules whose expression is frequently dysregulated in cancer cells;
3. Clinical-grade synthetic agonist molecules, such as aminobisphosphonates and PAg, that specifically induce activation, expansion and sensitization of human tumor cells;
4. Simple handling and elevated in/ex vivo expansion index;
5. Absence of alloreactivity (no MHC class I/II restrictions);
6. Capacity to reach and infiltrate tumors;
7. Direct and indirect cytotoxic activities against tumor cells, through the secretion of lytic molecules and pro-inflammatory cytokines.

## Successes and Limitations of Vγ9Vδ2 T Cell Cancer Immunotherapies

Several types of immunotherapies that aim at helping the immune system to better react against tumor cells, are used to treat cancer. They include immune checkpoint inhibitors, monoclonal antibodies and immune cell therapy. In this latter category, active and passive immunotherapies are distinguished, according to the approaches developed for inducing Vγ9Vδ2 T cell activation and expansion.

Regarding active immunotherapies, several strategies have been considered to obtain *in vivo* activation of Vγ9Vδ2 T cell effectors induced following administration(s) of specific clinical-grade agonist molecules, such as PAg or aminobisphophonates, together with pro-proliferating cytokines (e.g., IL-2) (32, 33). These approaches originated from initial observations describing increased frequencies of peripheral Vγ9Vδ2 T cells in hematological cancer patients treated with pamidronate (34). In patients with non-Hodgkin's lymphoma or multiple myeloma, systemic administrations of both pamidronate with IL-2 were tolerated by patients and induced expansions of endogenous peripheral Vγ9Vδ2 T cells, accompanied by partial remissions of cancer in some patients (35). Next, this strategy was applied to solid tumors (i.e., non-hormonal prostate cancer) and showed that activation of Vγ9Vδ2 T cells *in vivo* was associated with the development of a pro-inflammatory(IFN-γ) responses (36). Following these first encouraging results, several clinical trials have been conducted in patients with renal cell carcinoma or bone metastases deriving from breast or prostate cancers (32, 33). These studies have demonstrated therapeutic responses such as stabilized diseases and partial remissions in some patients (37–39). More recently, the efficacy of this strategy was improved in patients with malignant hemopathies receiving haploidentical donor lymphocyte infusion (40). Importantly, the majority of treated patients in these trials experienced mild side effects (i.e., flu-*like* syndrome), likely associated to IL-2, thus confirming the reduced toxicity of this strategy. To further improve its specificity, synthetic PAg compounds have been produced at a clinical grade (i.e., BrHPP) and tested *in vivo*. In metastatic renal cell carcinoma patients who received repeated infusions of BrHPP and IL-2, potent Vγ9Vδ2 T cell expansions with moderate clinical activities were observed (41). These phase I/0 clinical assays showed a satisfactory feasibility of this approach, with a reduced toxicity (mainly due to IL-2) and excellent Vγ9Vδ2 T cell expansion rates, but modest clinical efficacies, that might have been complicated by the bad clinical condition of cancer patients. Further studies, including assays in monkeys, next pointed out potential issues such as the progressive exhaustion of Vγ9Vδ2 T cells (i.e., tachyphylaxis), altered immune status, activation contexts and tissue homing, that should be solved to improve the therapeutic efficacy of this strategy in cancer treatment (42).

Passive immunotherapies, which are based on *ex vivo* PBL(*Peripheral Blood Lymphocytes*)-Vγ9Vδ2 T cell expansions and cell transfer(s) have been tested in renal carcinoma patients with reduced toxicity by low therapeutic efficacy (43). Next, several clinical trials have been carried out in patients with circulating or solid cancers, who have been treated by adoptive transfer of autologous Vγ9Vδ2 T cells, amplified *ex vivo*, associated with IL-2 and zoledronate (32, 33). In general, these studies indicated a good feasibility but a low, although promising, therapeutic efficacy of this therapy as evidenced by some partial/complete cancer remissions. Clinical trials of adoptive transfer of allogeneic Vγ9Vδ2 T cells are currently underway. Recently, a case report of a patient with a stage IV cholangiocarcinoma showing recurrent mediastinal lymph node metastasis after liver transplantation was published. This patient received consecutive infusions of allogeneic Vγ9Vδ2 T cells which have been expanded from PBMC (*Peripheral Blood Mononuclear Cells*) of a healthy donor. No adverse effects were detected and a significant clinical response, with no detectable peritoneal lymph node metastasis, was reported at the end of treatment (44). Improved strategies for redirecting immune cell effectors to target cells expressing γδ T cell tumor antigens are being generated and tested for therapy (45). Thanks to the significant advance of genetic engineering and transduction approaches, the efficient expression of natural or optimized TCR (e.g., γδ TCR) can be realized in lymphocytes (46). The clinical-grade production of these engineered effectors [e.g., CAR (*Chimeric Antigen Receptor*) γδ T cells] has recently been validated and is currently being tested in patients with leukemia or multiple myeloma (33, 46).

Though promising (e.g., feasibility, safety), the results of these clinical trials, though exploratory or phase 0/I only, stressed the urgent need of developing new Vγ9Vδ2 T cell-immunotherapies (e.g., combined therapies) and optimizing their use (e.g., clinical positioning) to significantly improve their clinical efficacy in circulating and solid oncological indications. The achievement of this ambitious goal should require the use of robust physiological preclinical models *in vivo* for assessing both the feasibility and efficacy parameters of these strategies.

## Implementation of Preclinical Cancer Models and Vγ9Vδ2 Immunotherapies

**Figure 1:**
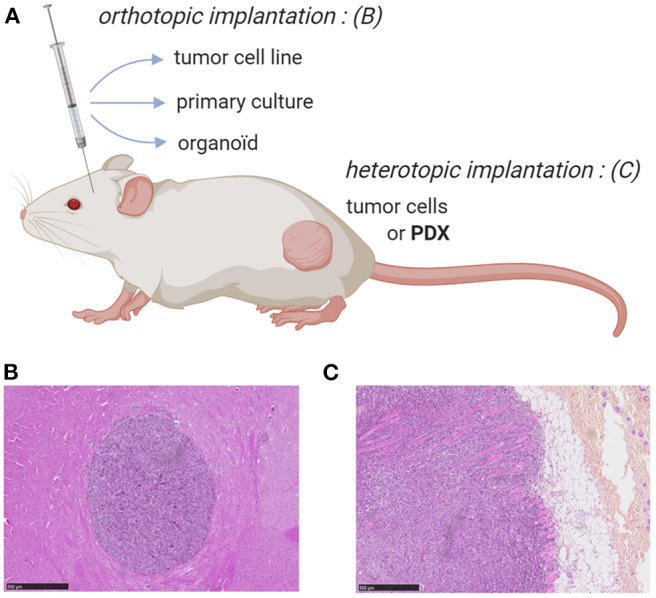
Mouse models for ortho- or heterotopic administrations of human tumor cells. **(A)** Schematic representation of human tumor cell implantation strategies in immunodeficient mouse (e.g., NSG). Human tumor cells from either cell lines, primary/organoid cultures or patient-derived xenograft can be implanted orthotopically or heterotopically, respectively. **(B,C)** Immunohistochemistry (hematoxylin and eosin stainings) of orthtopic (brain) implantation of cells from a primary culture of human glioblastoma (GBM-1) **(B)** and heterotopic (subcutaneous) implantation of human prostate cancer cells (PC-3 cell line) **(C)**. Bars: 500 μm. NJ & ES, unpublished work.

A substantial number of preclinical *in vivo* cancer models, including, for some of them, Vγ9Vδ2 T cell immunotherapies, have been carried out using heterotopic mouse models established with subcutaneous injections of cultured human tumor cell lines. Among various advantages, these approaches allow a fast and easy monitoring of tumor growth by physical measurement of tumor volume (47). However, these fast growing models, also generally used for non-Vγ9Vδ2 T cell therapies, rarely faithfully recapitulate the complexity and heterogeneity of human oncological disease (e.g., origin of tumor cells, nature of the environment), also because of the large number of cells implanted in this particular localization (48, 49). Another problem is linked to their limited ability to disseminate (e.g., metastasis). To address this problem, it is important to orthotopically implant tumor cells (i.e., corresponding anatomical position) and, if possible, in a minimal quantity to further mimic the first stages of tumor development and its natural dissemination, according to the organ of origin (Figure 1). Importantly, the growing tumor should be characterized using various approaches such as imaging, histology or analysis of the phenotype of tumor cells to accurately determine the relevance of this model *in vivo* (50–52).

An indisputable tool for creating preclinical murine models representative of human pathology, not to mention therapies subsequently used, is the graft of explanted tumor samples from cancer patients. Two main strategies have been proposed: (i) the administration of dissociated and cultured tumor cells or (ii) the implantation of tumor fragments (hereafter called PDX, *patient-derived xenograft*) (Figure 1). These human tumors can heterotypically or orthotopically engrafted in immunodeficient mice, such as NSG (*NOD.Cg-PrkdcscidIl2rgtm1Wjl/SzJl*), with engraftment rates (40–95%) related to the presence of stroma. In the case of dissociated and cultured primary tumor cells, all these preparation steps might affect their phenotype and promote the biased growth of particular tumor variants. This issue can be addressed using tumor fragments (53) but the implantation of such large PDX is rarely possible in orthotopic because of size or surgical issues (e.g., intracerebral glioblastoma) (Figure 1). The engraftment and the growth of PDX often take 2–4 months, which can vary by tumor type, implant location, and the strain of immunodeficient mice utilized. This long duration parameter, further complicated by the necessity to divide and to re-implant small fragments of grafted PDX, strongly limits the use of PDX models and its interest for establishing robust, homogeneous and reliable tumor mouse models (i.e., progressive loss of tumor heterogeneity and replacement of the human microenvironment disappears by a murine stroma) (53).

In the absence of relevant and robust *in vitro* tumor models, including the promising, but currently developed spheroid/organoid/3D tumor systems, the development of novel therapies still requires assessment steps in preclinical animal models before being proposed for therapy. Their use is expected to help predict selected therapeutic effects and analyze selected parameters such as feasibility, side effects and toxicity. In particular with regard to human Vγ9Vδ2 T cells, it is not possible to use syngeneic murine models due to the lack of counterpart of this lymphocyte subset in mice and its species specificity (4, 5). Mostly due to an elevated cost of care, non-human primates, which also contain PAg-reactive γδ T cells, are ill-developed for cancer therapy studies. Highly immunodeficient mice, such as nude or NSG mice, represent the most relevant strains of choice for developing human cancer and immunotherapy models. However, the downside is that most of these immunodeficient mouse models are not relevant for analyzing the contribution of the tumor environment and immune system components to the anti-tumor efficacy of immunotherapies. Rebuilding human immune system in mice remains possible, thanks to the systemic injection of human PBMC in irradiated mice (54). However, this approach might generate strong xenogeneic reaction against the host, which limits the relevance and the operational time window for using these humanized animal models. In addition, the management and reproducibility of these sophisticated models remains complicated. Right now, the mostly developed method of humanization remains the injection/grafting of human tumor cells into immunodeficient mice.

## Paths for the Development of Improved Vγ9Vδ2 Immunotherapies Using Preclinical Models

**Figure 2:**
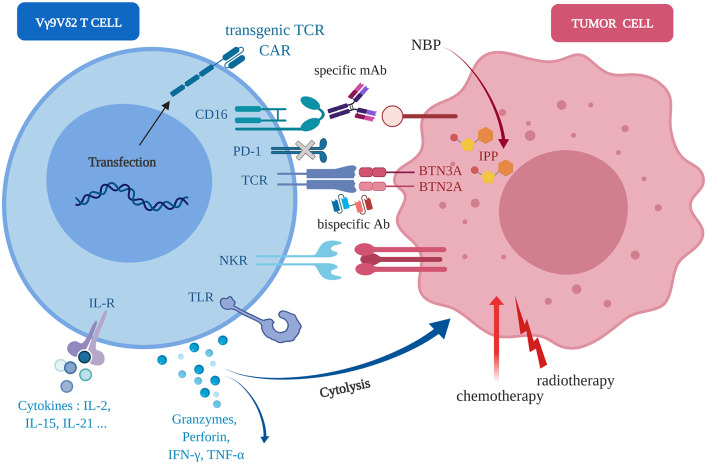
Molecular axes foroptimization(s) of anti-tumor Vγ9Vδ2 immunotherapies. Human Vγ9Vδ2 T cells (*left, blue*) can sense human tumor cells (*right, red*) of diverse tissular origins, in independent or cooperative manners, through various molecular pathways implicating wild-type or engineered TCR, NKR, FcR/CD16, TLR, CAR. Following activation, the direct (cytolysis) and indirect (e.g., pro-inflammatory cytokines release) anti-tumor reactivity might be enhanced by either agonist compounds, such as aminobisphosphonates (NBP), standard chemo-radiotherapies or environment factors, such as cytokines (e.g., IL-21).

Various preclinical strategies are currently developed to assess the therapeutic potential of novel optimized Vγ9Vδ2 immunotherapies targeting various pathways (which are summarized in Figure 2). In particular, several research and clinical groups aim at proposing immunotherapeutic strategies to enhance the reactivity and the cytolytic activity of human Vγ9Vδ2 T cells against tumor cells (16). Of note, the routes for treatment administration in preclinical models should be carefully selected. Most clinical treatments are either orally or systemically administered which might significantly contribute to an increased toxicity or alow therapeutic efficiency (e.g., low specificity). An increasing number of studies evidenced the benefits of local administrations for limiting the systemic toxicity while increasing therapeutic doses (55, 56). These principles should be applied, if clinically possible, for the adoptive transfer(s) of Vγ9Vδ2 T cells.

Following an initial assessment *in vitro*, both orthotopic and heterotopic preclinical models of human tumors from various types of tissues, have been implemented in mice and used for the following Vγ9Vδ2 T cell immunotherapy optimizations (Figure 2):

- Natural reactivity of allogeneic Vγ9Vδ2 T cells against some tumor cell types. For example, some human mesenchymal GBM cells naturally overexpress NKG2D ligands that induce a natural Vγ9Vδ2 T cell reactivity leading to the elimination of tumor cells *in vivo* (57);
- Transfer(s) of human Vγ9Vδ2 T cells combined to agonist compounds (e.g., aminobisphosphonate compounds) administration(s) to further increase the recognition of tumor cells by Vγ9Vδ2 T cells (47). These therapeutic components can be injected either systemically (e.g., intravenously) or close to the tumor sites (for solid tumors) in orthotopic models (50, 52);
- Transfer(s) of human Vγ9Vδ2 T cells combined to radio-chemotherapies. This strategy relies on the observations that these first-line standard treatments are proposed for most cancer indications and could promote cell stress events that could increase the reactivity of Vγ9Vδ2 T cells against human tumor cells (58–60);
- Transfer(s) of human Vγ9Vδ2 T cells combined to antibodies directed against inhibitory immune checkpoint molecules (e.g., PD-1) (61) or ADCC (*Antibody-Dependent Cellular Cytotoxicity*)-triggering molecules molecules (e.g., FcγRIIIA/CD16) (62, 63);
- Bispecific antibodies which are generated under various molecular formats and have a dual specificity for a selected tumor antigen (targeting) and for the Vγ9Vδ2 TCR (activation) (64).
- Agonist antibodies targeting Vγ9Vδ2 T cell activation molecules (e.g., ectodomain of BTN3A1) to induce a PAg-independent, but TCR-specific, reactivity of Vγ9Vδ2 T cells against tumor cells (65);
- Selected cytokines that can enhance the cytotoxic potential of Vγ9Vδ2 T cells, such as IL-21 (51, 66), or boost their proliferation during *ex vivo* amplification before adoptive transfer, such as IL-15 associated to Vitamin C (67).

## Concluding Remarks

In terms of phenotype and functions, the human Vγ9Vδ2 T cell subset represents a unique and highly attractive T cell population for designing efficient cancer immunotherapies. However, initial clinical trials targeting this subset yielded mixed results with both encouraging (e.g., good feasibility, weak toxicity) and disappointing observations (e.g., modest clinical efficacy), that could be attributed to inappropriately designed strategies or incorrect therapeutic settings. Nonetheless, these studies clearly met on the urgent need for proposing improved therapies, an objective that should be achieved through the development of more relevant and physiological preclinical models. Although first *in vivo* models have been proposed decades ago, with a major bias toward rodents (ie. mouse), their use for designing Vγ9Vδ2 T cell-immunotherapies has long been severely hampered by the strict species-specific restrictions of this primate subset. However, the research in this field widely accelerated in the past years thanks to the emergence and development of immunodeficient murine strains as well as improved grafting, mouse humanization and high-dimension detection/“big data” analysis approaches. Importantly, animal-free *in vitro* models (e.g., spheroids, organoids) are currently developed and should also be used soon in this process. Altogether, these elements should accelerate the entry of this research field within a novel dimension, which should be evidenced by an increased number of successful immunotherapeutic trials in cancer patients.

## Author Contributions

NJ and ES contributed to the writing process, prepared the manuscript, and approved the final version.

## Conflict of Interest

The authors declare that the research was conducted in the absence of any commercial or financial relationships that could be construed as a potential conflict of interest.
